# Atypical Trigeminal Neuralgia Secondary to Meningioma

**DOI:** 10.1155/2015/462569

**Published:** 2015-11-19

**Authors:** Premeshwar Niwant, Mukta Motwani, Sushil Naik

**Affiliations:** ^1^Department of Oral Medicine and Radiology, Sharad Pawar Dental College and Hospital, DMIMSU, Sawangi (Meghe), Wardha, India; ^2^Department of Oral Medicine and Radiology, Vidya Shikshan Prasarak Mandal Dental College & Research Center (VSPM DCRC), Nagpur, India; ^3^Department of Public Health Dentistry, Vidya Shikshan Prasarak Mandal Dental College & Research Center (VSPM DCRC), Nagpur, India

## Abstract

Trigeminal neuralgia is a disorder of the fifth cranial nerve that causes episodes of intense, stabbing, electric shock-like pain that lasts from few seconds to few minutes in the areas of the face where the branches of the nerve are distributed. More than one nerve branch can be affected by the disorder. We report an unusual case of trigeminal neuralgia affecting right side of face presenting atypical features of neuralgia and not responding to the usual course of treatment. The magnetic resonance imaging study of brain revealed a large extra-axial mass involving right cerebellopontine angle region causing moderate pressure effect on trigeminal nerve and brain stem. The aim of this case report is to show a tumor of cerebellopontine angle, presenting clinically as atypical trigeminal neuralgia.

## 1. Introduction

Trigeminal neuralgia (TN), also known as prosopalgia [1], suicide disease [2], or Fothergill's disease [3], is a neuropathic disorder characterized by episodes of intense pain in the face, originating from the branches of the trigeminal nerve. The clinical association between TN and hemi facial spasm is the so-called tic douloureux [4]. It has been described as one of the most painful conditions known to mankind [5]. It is estimated that 1 in 15,000 or 20,000 people suffers from TN, although the actual figure may be significantly higher due to frequent misdiagnosis [6].

Trigeminal neuralgia has typical clinical features and various theories have been put forward as to its etiology. It is usually associated with pathosis along the course of the nerve. Most of the times the cause is not known and trigeminal neuralgia is termed as idiopathic TN.

Anomaly of the superior cerebral artery causing demyelization and pressure of the aneurysms of the intrapetrous portion of the internal carotid artery causing irritation of the trigeminal ganglion leading to paroxysmal trigeminal neuralgia are recently blamed causes of idiopathic TN.

Many lesions such as trigeminal neuromas in the middle cranial or the posterior fossa, epidermoid tumors, Meckle's cave, arteriovenous malformations, aneurysms, and vascular compression have been suggested as the causes. It is also recognized that TN occurs in about 2–4% of patients with multiple sclerosis [7].

Classical trigeminal neuralgia [8] typically manifests as a sudden, unilateral, intermittent, paroxysmal, sharp shooting, lancinating, shock-like pain, elicited by slight touching superficial to the “trigger points” and pain radiates from that point, across the distribution of one or more branches of the trigeminal nerve.

Pain is always unilateral and does not cross the midline. The pain is of short duration and lasts from a few seconds to several minutes. Refractory period is also present between the attacks. Most of these patients respond to treatment with carbamazepine and this can be used as a diagnostic indicator in TN.

Many patients present with less classic symptoms and may mimic toothache, sinusitis, stomatitis, or other inflammatory conditions and therefore, for diagnosis of neuralgic pain, proper clinical examination along with thorough history is mandatory.

The neuralgic symptoms in younger age group (less than 35 yrs) should alert the clinician to a possible intracranial space occupying lesion or intracranial arteriovenous anomalies.

Lesions of cerebellopontine angle (CPA) are frequent and represent 6–10% of all intracranial tumors [9]. Acoustic neuromas, which are also called vestibular schwannomas and meningiomas, are the two most frequent lesions and account for approximately 85–90% of all cerebellopontine angle tumors [10].

In most cases, magnetic resonance imaging and computed tomography show typical features of acoustic neuromas or meningiomas and are sufficient to establish the diagnosis. Meningiomas are usually hemispheric, semilunar masses with a broad petrous base to which they are attached whereas acoustic neuromas are usually round or oval masses in the cerebellopontine cistern that emerge from the internal auditory canal and grow posteriorly because of the anterior limit represented by the cisternal segment of the facial nerve [11].

In this paper, we discuss the importance of investigating each and every case of TN for proper diagnosis before we term it to be idiopathic TN.

## 2. Case Report

A 40-year-old male patient reported to Outpatient Department of Vidya Shikshan Prasarak Mandal Dental College & Research Center (VSPM DCRC), Nagpur, with the chief complaint of pain in the right ear and preauricular region for 2 yrs. Patient had visited various dentists but did not get any relief. Patient was diagnosed as a case of glossopharyngeal neuralgia three months prior to his visit and was treated with tablets carbamazepine 100 mg thrice daily which then increased to 200 mg thrice daily but without any satisfactory relief. Severity and intensity of pain increase on the same side of face in spite of taking increased dose of carbamazepine.

When patient visited our department he had severe, intermittent, and lancinating pain in that region. Pain was paroxysmal and each episode lasted from few seconds to few minutes, with trigger points in the preauricular and postauricular region on right side of face. Patient also gave history of episodes of severe awakening pain during nights but no history of pain on deglutition or during movements of tongue. There was no history of seizures, nausea, vomiting, and vision changes. On examination, trigger zones were present on malar area, zygomatic buttress area, and also body and ramus of mandible but otherwise the patient's general condition was normal. The pains are bright and stimulating and are accurately located by the patient. The behavioral characteristics of the pain are neurogenous.

All these findings were suggestive of trigeminal neuralgia but as the patient was not responding to the usual course of treatment and the severity of pain increases, an intracranial pathology was suspected in this case. Magnetic resonance imaging was advised which revealed an intracranial tumor.

**Figure 1:**
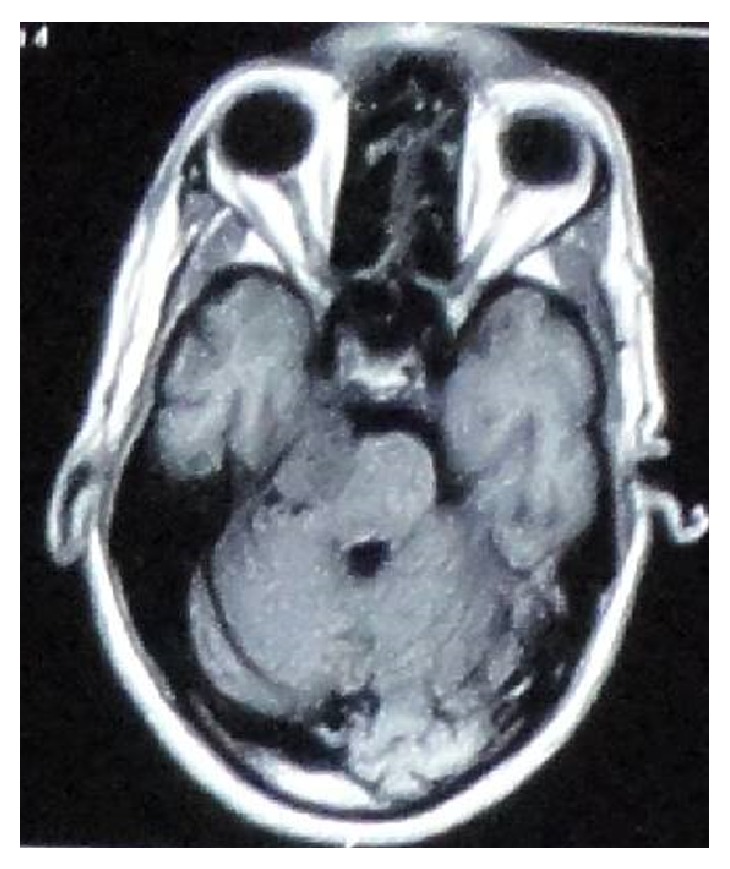
Axial view of T1W image showing isointense, well-defined lesion seen in right cerebellopontine angle causing rotation of brain stem and compression of contralateral CP angle.

**Figure 2:**
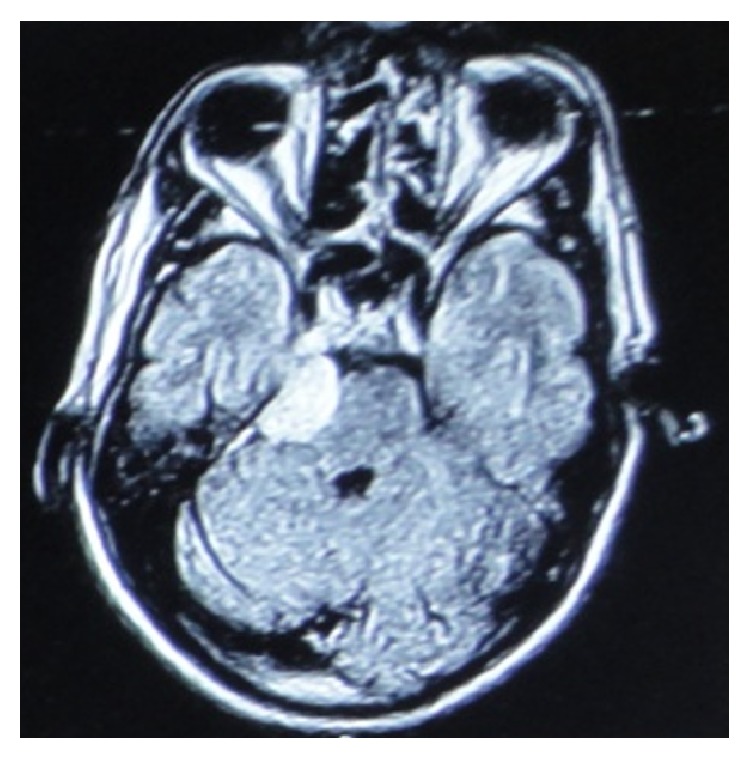
Flair axial image showing hyperintense, well-defined lesion seen in right cerebellopontine angle causing rotation of brain stem and compression of contralateral CP angle.

Magnetic resonance imaging (MRI) showed well-defined intensity enhancing extra-axial mass involving right cerebral pontine angle region measuring 2.5 × 2.0 × 1.8 cm in anteroposterior, transverse, and superoinferior direction, respectively, and causing moderate mass effect on trigeminal nerve and brain stem (Figures 1 and 2).

**Figure 3:**
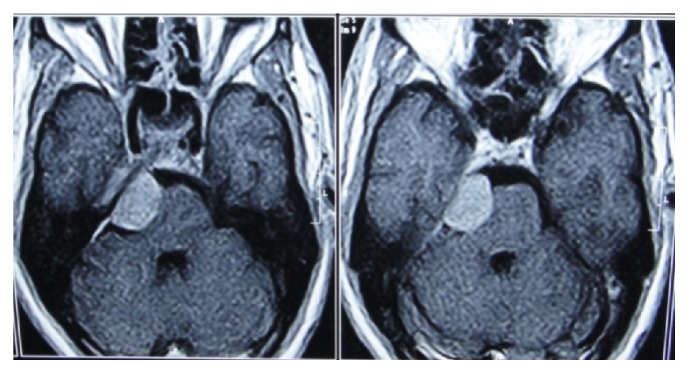
MRI T1W axial image with contrast shows well-defined intensity enhancing extra-axial mass lesion involving right cerebral pontine angle region. MRI impression: MRI study in contrast revealed possibility of CP angle Schwannoma or Meningioma.

**Figure 4:**
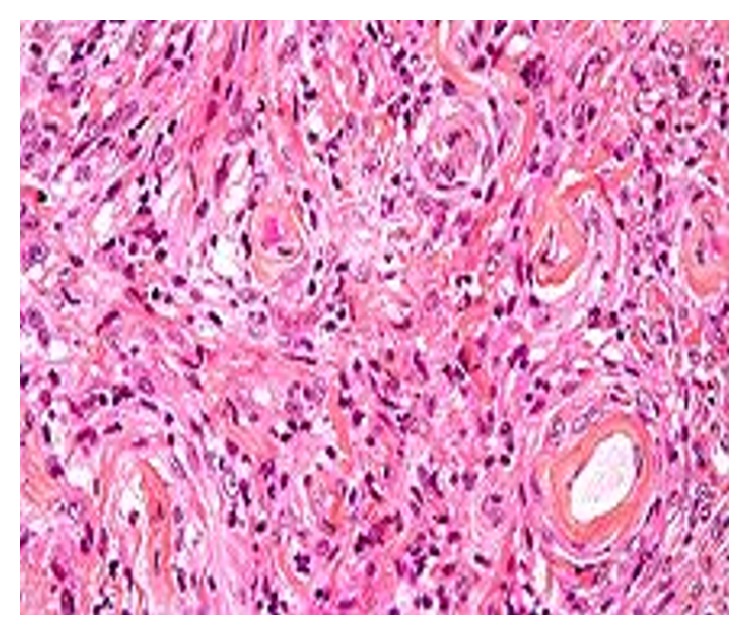
Histopathology slide: histopathologically showing characteristic whorling suggestive of meningioma.

Further MRI with contrast revealed possibility of cerebellopontine angle Schwannoma or meningioma (Figure 3). The patient was referred to a neurologist for further management. The tumor was surgically removed at Bombay Hospital, Mumbai, under general anesthesia. Histopathological examination showed characteristic whirling suggestive of meningioma (Figure 4). Final diagnosis of trigeminal neuralgia involving maxillary and mandibular nerve secondary to meningioma was confirmed.

## 3. Discussion

It is estimated that 15% of patients with trigeminal neuralgia have intracranial pathology that compresses the trigeminal nerve. This often occurs in the cerebellopontine angle, pons, and medulla from where the trigeminal nerve exits. Failure to properly assess for secondary TN is a major potential pitfall. So a careful examination of all the cranial nerves along with MRI of the brain is advisable in patients younger than 60 yrs., so as to identify the presence of structural lesions like tumor, cerebral aneurism, acoustic neuroma, and so forth.

One should also lookout for symptoms related to compression of surrounding tissues, which can also affect other cranial nerves and blood vessels.

The typical features of meningiomas are seizures, headaches, nausea, vomiting, vision, and behavioral and cognitive changes. Sometimes no symptoms occur and tumor is detected incidentally [12]. In this case, no typical symptoms of meningioma were seen; it rather presented as a typical case of trigeminal neuralgia.

Various treatment modalities are available for management of trigeminal neuralgia which has been tried for many years. Medicinal therapy is usually given for the treatment of TN. Surgical intervention can be attempted only if and when the pharmacologic therapy fails. Carbamazepine is the drug of choice for TN. 100 mg tablet thrice daily usually resolves pain of TN.

Side effects of this drug may cause bone marrow depression, drowsiness, blurred vision, and vertigo. Therefore, monitoring the bone marrow activity by obtaining the complete blood count prior to initiating therapy and routinely thereafter is indicated.

Anticonvulsant medications pose risk of sedation and ataxia particularly in elderly patients, which may make driving or operating machinery hazardous. So patients should avoid maneuvers that trigger pain. Many times the diagnosis is not established and the clinician goes for multiple dental extractions which are of no use even if pain radiates into the gums. Patients also need to understand that medications for TN are only palliative and often are of limited and temporary value. If the patient does not respond at least partially to carbamazepine, we should suspect for idiopathic TN.

Tumor arising in the CPA or the tumor of the base of the skull or brainstem encroaching upon the CPA can produce TN like symptoms. The other associated symptoms of these tumors depend on the direction of growth of these tumors. In our case although the lesion arose from CPA, it did not have any feature of this tumor and rather it presented as atypical TN; therefore, diagnosis was difficult.

As the tumor grows upward into the superior aspect of the cerebellopontine angle, it encroaches upon the trigeminal nerve, producing a gradual decrease of the corneal reflex and facial analgesia and anaesthesia. Downward growth of these lesions results in hoarseness, numbness of the throat, or complaints of difficulty in swallowing. As with acoustic tumors, large meningiomas can produce cerebellar symptoms and signs or hydrocephalus with increased intracranial pressure [12].

Patient must be informed thoroughly of the risks involved with neurosurgical interventions, including anesthesia dolorosa that results from injury to the system in an attempt to treat trigeminal neuralgia. Numbness of the face is a constant part, which has also been referred to as trigeminal deafferentation pain. It results from damage to peripheral or central sensory pathways leading to partial or total loss of sensory nerve supply to a region of the body. Deafferentation pain is usually severe in intensity and highly resistant to treatment. It can spread to other orofacial structures by extension of the receptive field.

Some symptoms such as hypoesthesia, paresthesia or anesthesia, or hyperalgesia may be felt in the region innervated by the injured nerve and these symptoms may persist indefinitely [13].

## 4. Conclusion

The present case report focuses on the fact that the eyes cannot see what the mind does not know. It is not always possible to determine what causes trigeminal pain. However, several possibilities exist. The present case shows an unusual lesion of cerebellopontine angle tumor, rare cause of atypical TN, emphasizing the importance of investigating each and every case of trigeminal neuralgia for proper clinical diagnosis. As we cannot establish a relation between pathology in the brain or brainstem and a clinical picture in a patient, imaging technique preferably an MRI scan with a focus on the course of the trigeminal nerve can be of utmost importance for the diagnosis of such cases. Follow-up scans are needed because meningioma can recur years or even decades after treatment.
